# Using machine learning-based binary classifiers for predicting organizational members’ user satisfaction with collaboration software

**DOI:** 10.7717/peerj-cs.1481

**Published:** 2023-07-17

**Authors:** Yituo Feng, Jungryeol Park

**Affiliations:** 1 Management Information System, Chungbuk National University, Cheongju, South Korea; 2 Technology Policy Research Division, Electronics and Telecommunications Research Institute, Daejeon, South Korea

**Keywords:** Collaboration software, User satisfaction, Machine learning, Binary classifier, Prediction model, Feature importance

## Abstract

**Background:**

In today’s digital economy, enterprises are adopting collaboration software to facilitate digital transformation. However, if employees are not satisfied with the collaboration software, it can hinder enterprises from achieving the expected benefits. Although existing literature has contributed to user satisfaction after the introduction of collaboration software, there are gaps in predicting user satisfaction before its implementation. To address this gap, this study offers a machine learning-based forecasting method.

**Methods:**

We utilized national public data provided by the national information society agency of South Korea. To enable the data to be used in a machine learning-based binary classifier, we discretized the predictor variable. We then validated the effectiveness of our prediction model by calculating feature importance scores and prediction accuracy.

**Results:**

We identified 10 key factors that can predict user satisfaction. Furthermore, our analysis indicated that the naive Bayes (NB) classifier achieved the highest prediction accuracy rate of 0.780, followed by logistic regression (LR) at 0.767, extreme gradient boosting (XGBoost) at 0.744, support vector machine (SVM) at 0.744, K-nearest neighbor (KNN) at 0.707, and decision tree (DT) at 0.637.

**Conclusions:**

This research identifies essential indicators that can predict user satisfaction with collaboration software across four levels: institutional guidance, information and communication technology (ICT) environment, company culture, and demographics. Enterprises can use this information to evaluate their current collaboration status and develop strategies for introducing collaboration software. Furthermore, this study presents a novel approach to predicting user satisfaction and confirm the effectiveness of the machine learning-based prediction method proposed in this study, adding to the existing knowledge on the subject.

## Introduction

In today’s digital economy, collaboration software has emerged as a critical tool for organizations seeking to enhance productivity, communication, and innovation among their workforces (Soto-Acosta, 2020; Vial, 2021). The COVID-19 pandemic has further driven the demand for collaboration software, with the market in South Korea expected to reach 9,103.7 billion won by 2026 (Markets & Markets, 2021). While the benefits of collaboration software are well-documented, research has shown that it can only produce positive results when organizational members are satisfied with using it (Guinan, Parise & Rollag, 2014; Mäntymäki & Riemer, 2016; Waizenegger et al., 2020).

Numerous scholars have investigated factors affecting satisfaction with collaboration software, aiming to optimize its implementation and use (Lee, 2017; Fu, Sawang & Sun, 2019; Johnson, Zimmermann & Bird, 2021). A comprehensive analysis of the relevant literature was conducted, and the findings have been summarized in Table 1. In recent years, the literature on improving user satisfaction with collaboration software has predominantly focused on four key aspects: (1) theoretical frameworks and models, in which articles develop theoretical frameworks and models to understand user satisfaction in the context of collaboration software; (2) factors influencing user satisfaction, where articles explore the factors that impact user satisfaction, such as usability, user experience, customization, and support; (3) evaluation and measurement of user satisfaction, with articles concentrating on methods and approaches to evaluate and measure user satisfaction with collaboration software; and (4) best practices and strategies, where articles discuss best practices and strategies for organizations to enhance user satisfaction with collaboration software.

**Table 1:** Literature review.

Main categories	Author(s) & Year	Objective/Focus
Theoretical frameworks and models	Feng, Park & Feng (2023)	These articles focus on developing theoretical frameworks and models to understand user satisfaction in the context of collaboration software.
	Strode, Dingsøyr & Lindsjorn (2022)	
	Kuruzovich et al. (2021)	
	Yao et al. (2020)	
Factors influencing user satisfaction	Tea et al. (2022)	These articles investigate the factors that impact user satisfaction, such as usability, user experience, customization, and support.
	Tarun (2019)	
	Zamani & Gum (2019)	
Evaluation and measurement of user satisfaction	Chen et al. (2020)	These articles focus on methods and approaches to evaluate and measure user satisfaction with collaboration software.
	Salam & Farooq (2020)	
	Shonfeld & Magen-Nagar (2020)	
	Karlinsky-Shichor & Zviran (2016)	
Best practices and strategies	Sangwan, Jablokow & DeFranco (2020)	These articles discuss best practices and strategies for organizations to improve user satisfaction with collaboration software.
	Gil et al. (2016)	
	Mistrík et al. (2010)	

A comprehensive analysis of the relevant literature reveals that the majority of existing studies focus on examining strategies organizations can use to enhance user satisfaction after the implementation of collaboration software. Although these studies provide valuable insights into employees’ experiences with collaboration software after its introduction, a gap exists in predicting user satisfaction prior to implementation.

Neglecting to forecast future user satisfaction can lead to several adverse consequences for organizations: (1) Reactive approach: Assessing user satisfaction after introducing collaboration software constitutes a reactive approach. Consequently, organizations can only address issues after they have already affected employees, potentially leading to a longer period of reduced productivity and increased frustration as employees struggle to adapt to the new system (Baah et al., 2020). (2) Higher implementation costs: Addressing dissatisfaction post-implementation may require additional investments in software customization, training, or even replacement. These costs can be significant and could have been avoided with a proactive approach to predicting user satisfaction before implementation (Meske & Stieglitz, 2013). (3) Resistance to change and decreased adoption: IF employees encounter issues with collaboration software after its introduction, they may become more resistant to adopting the new tool or process. This resistance can slow the integration of the software into daily workflows, reducing the potential benefits and efficiencies the software was intended to provide (Berger & Thomas, 2011). (4) Employee turnover and dissatisfaction: Addressing user satisfaction only after introducing collaboration software may cause employees to become disillusioned or frustrated with the organization’s technology choices. This dissatisfaction can contribute to higher employee turnover rates, which can be costly and detrimental to overall organizational success (Sageer, Rafat & Agarwal, 2012). (5) Missed opportunities for optimization: Predicting user satisfaction before introducing collaboration software allows organizations to identify potential areas for improvement in the software’s design, features, or usability. By proactively addressing these issues, organizations can ensure that the software is better tailored to their employees’ needs, leading to more efficient workflows and higher overall satisfaction (Boehm, 2011).

Therefore, this article aims to address the gap in the literature by exploring the use of machine learning-based binary classifiers for predicting user satisfaction with collaboration software before implementation. By adopting a proactive approach and considering the potential impact of collaboration software on employee satisfaction before introducing it, organizations can make more informed decisions, optimize their software investments, and ultimately foster a more effective and harmonious work environment.

We will provide a detailed description of the case studies and methods used for predicting user satisfaction, evaluating their prediction accuracy to identify the classifier with the highest performance. The results of this study will contribute to the existing body of knowledge on collaboration software and user satisfaction and offer practical implications for organizations looking to implement such tools.

### Previous literature on predictive research using machine learning

Machine learning-based binary classifier is a method that classifies a set of elements into two categories using classification rules (Read et al., 2021). In the context of artificial intelligence, more and more scholars have conducted research related to prediction using machine learning-based binary classifiers (Khandani, Kim & Lo, 2010; Fu, Sawang & Sun, 2019; Dastile, Celik & Potsane, 2020; Yoo & Rho, 2020; Ho, Cheong & Weldon, 2021; Cocco, Tonelli & Marchesi, 2021; Naeem et al., 2021; Park, Kwon & Jeong, 2023). For instance, doctors predict the health of diabetes and cancer patients using random forest classifiers and NB classifiers. Practitioners use case datasets and similar disease features to classify and predict future patient health (Fu, Sawang & Sun, 2019). Moreover, scholars use machine learning-based binary classifiers to perform statistics and construct prediction methods to provide appropriate strategies for combating and managing the spread of epidemics like COVID-19 (Naeem et al., 2021). In the financial industry, people can predict Bitcoin prices through a framework based on a machine learning-based binary classifier and provide trading strategies for industry practitioners (Cocco, Tonelli & Marchesi, 2021). Bank lenders can use machine learning to analyze data from past loan officers and build credit risk prediction models. Predictive models can determine future loan applicants’ repayment ability and help banks decide whether to lend and reduce losses (Khandani, Kim & Lo, 2010; Dastile, Celik & Potsane, 2020). In education-related research, machine learning was used to construct a method to predict teacher job satisfaction and student satisfaction in remote learning during the COVID-19 pandemic (Yoo & Rho, 2020; Ho, Cheong & Weldon, 2021).

Overall, machine learning-based binary classifiers are highly feasible and superior for prediction due to their ability to use both categorical and numerical predictors and evaluate the importance of each predictor.

## Materials and Methods

**Figure 1:**
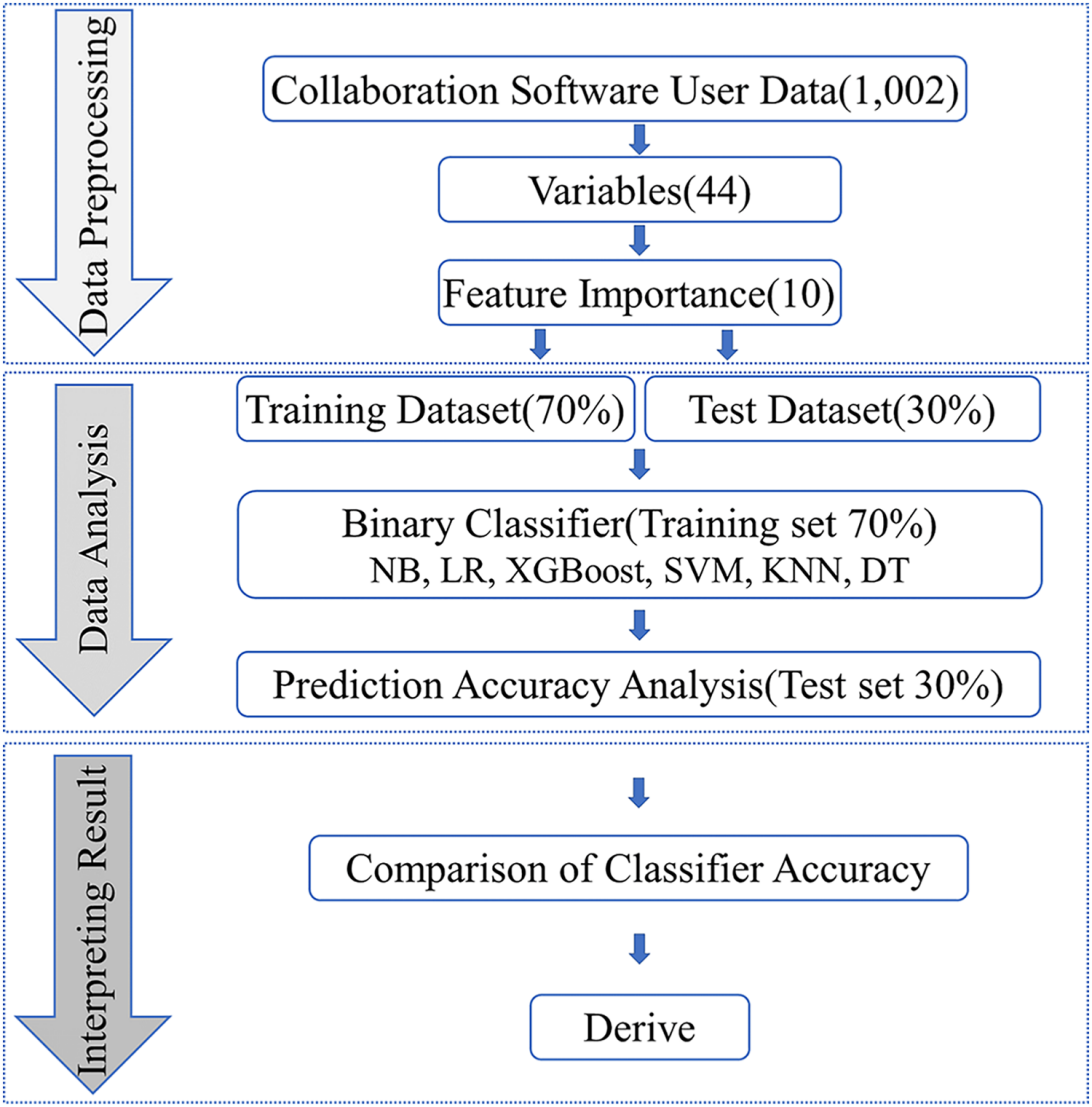
Research study protocol.

Our research aims to develop a machine learning-based approach for predicting employee satisfaction with collaboration software, thereby providing enterprises with valuable insights and effective management strategies for implementing and utilizing such software. To achieve this, we employed public data with high reliability and an extensive sample size for our investigation. The research process comprises three stages, as illustrated in Fig. 1: data preprocessing, data analysis, and interpreting result.

### Data source

This study utilized publicly available data from the “Smart Work Fact-Finding Report 2020” conducted by the Korea National Information Society Agency. The report (Approval Number: NIA III-RSE-A-20010) is based on an online survey of 1,900 employees from September 1 to September 30, 2020, across 17 metropolitan cities and regions in South Korea. The survey questionnaire covered a range of topics, including perceptions of smart work, the status of smart work, the work environment, the effects of smart work, obstacles to smart work, government support measures, and respondent information. Table 2 provides an overview of the questionnaire’s content.

**Table 2:** Survey content of questionnaire.

Sortation	Measurement items
PART A Perceptions about smart work	How much did you know about smart work before this study?
	How much do you think smart work is necessary?
PART B Smart work usage status	What kind of smart work do you currently use to perform your work?
	When did you first use smart work?
	Why do you use smart work?
	Why do you not use smart work?
	How often do you use smart work?
PART C Smart work-based environment	ICT environment, willingness to adopt smart work, and organizational culture.
	What business infrastructure is your company using?
	If working from home, what is the basic environment you can use?
PART D The effect of smart work	After using smart work, how much do you think it was helpful?
	How satisfied are you with the collaboration software you used?
PART E	Smart work’s obstacles and government support measures
PART F	Respondent information

The data used in this study are publicly available and can be accessed by anyone who applies. The Korea National Information Society Agency provides users with reusable information and allows for both for-profit and non-profit use. Data used in this study can be obtained by applying at (https://www.nia.or.kr/site/nia_kor/03/10303040200002016092710.jsp).

Since the focus of this study is on predicting user satisfaction with collaboration software, only data related to practitioners who use collaboration software for smart work was used for predictive analysis, according to the type of smart work in the public data. The dataset included a total of 1,002 observations related to the use of collaboration software. The demographic characteristics of the participants are presented in Table 3.

**Table 3:** Demographic characteristics of collaboration software users.

Variables	Category	*n*	%
**Total**		1,002	100.00%
Gender	Male	627	62.60%
	Female	375	37.40%
Age (years)	$\leq$35	227	22.66%
	36–45	348	34.73%
	46–55	239	23.85%
	$\geq$56	188	18.77%
Type of business	Manufacturing	150	14.97%
	Construction	95	9.48%
	Wholesale and retail	124	12.38%
	Transport business	50	4.99%
	Accommodation and restaurant business	52	5.19%
	Publishing, video, broadcasting communication, information service business	79	7.88%
	Finance and insurance	73	7.29%
	Real estate business and rental business	42	4.19%
	Professional science and technology service industry	95	9.48%
	Education service industry	108	10.78%
	Health industry and social welfare service industry	94	9.38%
	Associations and other personal service businesses	40	3.99%
Company size	1–19	291	29.00%
	20–99	261	26.00%
	100–299	153	15.20%
	300–499	86	8.50%
	More than 500	211	21.30%

### Predictor variable

In this study, we used public data that consisted of typical Likert items with five response options: strongly disagree, disagree, neither agree nor disagree, agree, and strongly agree. To improve prediction accuracy, we discretized the predictor variable of user satisfaction. This variable is derived from the question item “How satisfied are you with the collaboration software you used?” in Table 2, PART D.

We created a discrete predictor variable from the mean of user satisfaction, assigning values of 0 and 1 to represent low and high user satisfaction, respectively. Out of the 1,002 participants who used collaboration software, 401 (40.02%) reported low user satisfaction, while 601 (59.98%) reported high user satisfaction. Discretization is a crucial step in machine learning-based research, as it simplifies data representation and enhances understanding (Liu et al., 2002; Tsai & Chen, 2019).

### Explanatory variable

To enhance prediction accuracy, we employed XGBoost’s feature importance algorithm to identify the most crucial explanatory variables for our predictive model. Feature importance algorithms enable the elimination of redundant and irrelevant features, thereby improving the performance of classifiers (Jiang et al., 2022).

This specific process is depicted in the first part of Fig. 1. We initially removed variables related to personally identifiable information and missing data from the original 1,002 datasets, yielding 44 explanatory variables. Subsequently, we utilized XGBoost’s algorithm to calculate the importance scores of these 44 explanatory variables, enabling us to pinpoint the most critical factors influencing prediction outcomes. Feature importance is assessed by the information gain before and after splitting a DT based on a particular feature. The more a feature can reduce the uncertainty in predicting the target variable, the greater its importance (Li et al., 2021). From the 44 explanatory variables, we selected the top 10 based on their importance scores for subsequent prediction and classification accuracy computations.

### Prediction model

To identify the most effective binary classifier for predicting user satisfaction with collaboration software, we compared the accuracy of several mainstream machine learning-based binary classifiers. The classifiers evaluated in this study included the NB, LR, XGBoost, SVM, KNN, and DT. By analyzing the performance of these classifiers, we were able to determine the most accurate and effective approach for predicting user satisfaction with collaboration software.

The data analysis phase of the research process, depicted in Fig. 1, outlines the accuracy prediction process. We divided the original data into a 70% training set and a 30% test set. The binary classifier first trains using the training set, and then it analyzes the accuracy using the test set.

In previous studies, researchers employed various accuracy metrics such as accuracy, precision, recall, and F1-score, depending on their experimental objectives. Although metrics for judging classifier performance are a crucial issue in machine learning, there is no broad consensus on a unified standard (Chicco & Jurman, 2020). In this study, we use accuracy, the most popular metric in binary classifier tasks, to judge the performance of the binary classifiers. Accuracy is the most intuitive performance measure, and it is simply a ratio of correctly predicted observations to the total observations (Mali et al., 2022). The formula for accuracy is shown below.

$\left( {{\rm Accuracy}} \right) = {\rm \; }\displaystyle{{TP + TN} \over {TP + FN + FP + TN}}$

where:

– True positives (TP) are the instances that were correctly classified as positive by the model.

– True negatives (TN) are the instances that were correctly classified as negative by the model.

– False positives (FP) are the instances that were incorrectly classified as positive by the model when they were actually negative.

– False negatives (FN) are the instances that were incorrectly classified as negative by the model when they were actually positive.

The accuracy rate ranges from 0 to 1, where 0 indicates that none of the instances were classified correctly, and 1 indicates that all instances were classified correctly. A higher accuracy rate indicates better predictive performance of the model.

### NB classifier

The NB classifier is a widely adopted machine learning technique rooted in Bayes’ theorem, used primarily for classification problems. Despite its simple nature, it excels in handling extensive datasets and has been successfully applied in various fields such as text classification, spam detection, and sentiment analysis. The term “naive” stems from the algorithm’s core assumption that all features are conditionally independent of each other given the class label—an assumption that may not always hold true in real-world scenarios (Sun, Li & Fan, 2021).

Nonetheless, the naive Bayes classifier has consistently demonstrated strong performance in diverse forecasting and prediction tasks, thanks to its straightforward nature, computational efficiency, and ease of implementation. The algorithm computes the posterior probability of each class label considering the feature values and subsequently assigns the instance to the class with the highest probability. In specialized forecasting processes, the naive Bayes classifier can offer valuable predictions by employing its probabilistic approach to estimate the likelihood of various outcomes based on available data.

In conclusion, the naive Bayes classifier is an effective and resource-efficient machine learning algorithm for tackling classification and forecasting challenges. While it relies on the simplifying assumption of feature independence, it has proven to be remarkably effective in a range of applications, such as text classification, spam filtering, and sentiment analysis. The algorithm’s probabilistic nature enables it to make reliable predictions in specialized forecasting processes by gauging the likelihood of different outcomes according to the input data.

The formula for Bayes’ theorem is:

${\rm P}\left( {{\rm C|X}} \right) = \displaystyle{{{\rm P}\left( {\rm C} \right){\rm P}\left( {{\rm X|C}} \right)} \over {{\rm P}\left( {\rm X} \right)}}\;$

where:

$\rm P(C|X)$ is the posterior probability of class C given the feature set X.

$\rm P(X|C)$ is the likelihood of observing feature set X given class C.

P(C) is the prior probability of class C.

P(X) is the probability of observing the feature set X.

In the naive Bayes classifier, the assumption is made that all features are conditionally independent given the class label. Thus, the likelihood term $\rm P(X|C)$ can be calculated as the product of the probabilities of each feature given the class:

$\rm P(X|C) = P(x1|C) * P(x2|C) * \ldots * P(xn|C)$

For each class, the classifier calculates the posterior probability $\rm P(C|X)$ and assigns the instance to the class with the highest probability.

### LR

LR is a widely used statistical method and machine learning algorithm for predicting the probability of an event occurring based on one or more predictor variables. It is particularly suitable for binary classification problems, where the outcome has two possible classes. The technique has been employed in various fields, including medicine, social sciences, and economics, to model the relationship between a binary dependent variable and one or more independent variables, which can be either continuous or categorical (Friedman, Hastie & Tibshirani, 2000; Lever, Krzywinski & Altman, 2016).

The primary concept behind LR is to model the probability of the event of interest (*e.g*., class 1) by fitting a logistic function to the predictor variables. The logistic function, also known as the sigmoid function, maps any real-valued input to a probability value between 0 and 1. The logistic function is given by:

$\rm P(Y=1|X) = 1 / \left (1 + e^{(-z)}\right)$

where:

– $\rm P(Y=1|X)$ is the probability of the event of interest (class 1) given the predictor variables X.

– z is the linear combination of predictor variables, represented as ${\rm{z}} = {\rm{\beta }}0 + {\rm{\beta }}1 \times 1 + {\rm{\beta }}2 \times 2 +  \ldots  + {\rm{\beta n}} \times {\rm{n}}$.

– β0, β1, …, βn are the regression coefficients that need to be estimated.

The estimation of the regression coefficients is typically done using the maximum likelihood estimation (MLE) method. Once the coefficients are estimated, the logistic function can be used to predict the probability of the event of interest for new instances.

In summary, LR is a widely employed statistical method and machine learning algorithm for binary classification problems that models the probability of an event using a logistic function. The algorithm estimates the regression coefficients by fitting the logistic function to the predictor variables and uses the resulting function to predict the probability of the event of interest for new instances.

### XGBoost

XGBoost, short for eXtreme gradient boosting, is a powerful and efficient machine learning algorithm designed for solving classification, regression, and ranking problems. It is an extension of the gradient boosting algorithm, which combines the strengths of multiple weak learners, typically decision trees, to create a more accurate and robust model (Atef, Elzanfaly & Ouf, 2022). XGBoost has gained widespread popularity due to its superior performance, scalability, and versatility across a variety of domains, including finance, healthcare, and natural language processing.

The core principle behind XGBoost is the iterative process of building a strong learner by optimizing an objective function that comprises a loss function and a regularization term. The loss function measures the difference between the predicted and true outcomes, while the regularization term prevents overfitting by penalizing complex models. XGBoost employs gradient boosting, which is an additive model that updates the weak learners by minimizing the loss function using gradient descent.

The specific forecasting process using XGBoost involves the following steps:

1) Initialize the model with a constant value or a simple model that minimizes the objective function. This serves as the base model.
2) For each iteration (t = 1, 2, …, T):
  - Compute the gradient and Hessian of the objective function with respect to the current model's predictions. These values indicate the direction and magnitude of change needed to minimize the objective function.
  - Build a new decision tree to fit the gradient and Hessian, which approximates the optimal structure for minimizing the objective function.
  - Determine the optimal step size (learning rate) and update the model by combining the base model and the new decision tree.
  - Regularize the model to control complexity and prevent overfitting.
3) Combine the base model with the results from all iterations to produce the final prediction.

The XGBoost algorithm can be represented mathematically using the following formula: ${{\rm{F}}_{\rm{t}}}({\rm{x}}) = {{\rm{F}}_{{\rm{t}} - 1}}({\rm{x}}) + {\rm{\eta }} * {{\rm{h}}_{\rm{t}}}({\rm{x}})$

Here, $\rm F_t(x)$ denotes the prediction at iteration t, $\rm F_{t-1}(x)$ represents the previous prediction, ${\rm{\eta }}$ is the learning rate, and $h_t(x)$ is the new decision tree that fits the gradient and Hessian.

In conclusion, XGBoost is a powerful and versatile machine learning algorithm with numerous advantages, including efficiency, scalability, and the ability to handle missing values. By incorporating gradient boosting techniques, regularization, and a specific forecasting process, XGBoost can effectively address various prediction tasks.

### SVM

SVM is a widely used supervised machine learning algorithm designed for classification and regression tasks. Its decision boundary is the maximum-margin hyperplane that solves the learning sample (Fu, Sawang & Sun, 2019). It has been successfully applied in various domains, such as bioinformatics, finance, and image recognition, due to its ability to handle linear and non-linear problems efficiently and accurately.

The key idea behind SVM is to find the optimal hyperplane that separates the data points of different classes with the maximum margin. The margin is defined as the distance between the hyperplane and the closest data points from each class, known as support vectors. A larger margin signifies a better separation between classes, resulting in a more accurate and robust model.

For linearly separable data, SVM constructs a linear hyperplane that can perfectly separate the classes. However, in cases where the data is not linearly separable, SVM employs the kernel trick to transform the input data into a higher-dimensional space, where a linear separation is possible. Commonly used kernel functions include linear, polynomial, radial basis function (RBF), and sigmoid kernels.

SVM is known for its robustness against overfitting, especially in high-dimensional spaces, as it only considers support vectors for model construction. Additionally, the algorithm allows for fine-tuning of model complexity through the use of hyperparameters, such as the cost parameter C and the kernel-specific parameters.

In summary, support vector machine is a versatile and efficient supervised machine learning algorithm suitable for classification and regression tasks. By finding the optimal hyperplane that maximizes the margin between classes, SVM can handle linear and non-linear problems effectively. Its robustness against overfitting and flexibility in adjusting model complexity through hyperparameters make it a popular choice among data scientists and machine learning practitioners.

The primary optimization problem for SVM in its primal form can be written as:

$$\matrix{
   {} \hfill & {{\rm{minimize}}{\mkern 1mu} 1/2{\mkern 1mu} \parallel {\rm{w}}{\parallel ^2} + {\rm{C}}\mathop \sum \nolimits^ \left( {{\xi _{\rm{i}}}} \right)} \hfill  \cr 
   {} \hfill & {{\rm{subject}}\>{\rm{to}}\>{{\rm{y}}_{\rm{i}}}({\rm{w}} \cdot {{\rm{x}}_{\rm{i}}} + {\rm{b}}) \ge 1 - {\xi _{\rm{i}}}\>{\rm{and}}\>{\xi _{\rm{i}}} \ge 0\>{\rm{for}}\>{\rm{i}} = 1,{\mkern 1mu}  \ldots ,{\mkern 1mu} {\rm{n}}} \hfill  \cr 

 } $$

where:

– w is the weight vector, which is orthogonal to the hyperplane.

– $\rm x_i$ are the data points.

– $\rm y_i$ are the class labels, either −1 or 1.

– b is the bias term.

– $\rm \xi_i$ are the slack variables, which allow for some misclassification in non-linearly separable cases.

– C is the cost parameter, a user-defined parameter that controls the trade-off between maximizing the margin and minimizing the classification error.

In the dual form, the optimization problem becomes:

$$\matrix{
   {} \hfill & {{\rm{maximize}}\mathop \sum \nolimits^ \left( {{{\rm{\alpha }}_{\rm{i}}}} \right)\, - \,1/2\,\mathop \sum \nolimits^ \left( {\mathop \sum \nolimits^ \left( {{{\rm{\alpha }}_{\rm{i}}}{{\rm{\alpha }}_{\rm{j}}}{{\rm{y}}_{\rm{i}}}{{\rm{y}}_{\rm{j}}}{\rm{K}}\left( {{{\rm{x}}_{\rm{i}}},\,{{\rm{y}}_{\rm{i}}}} \right)} \right)} \right)} \hfill  \cr 
   {} \hfill & {{\rm{subject}}\>{\rm{to}}\>0\, \le {{\rm{\alpha }}_{\rm{i}}} \le {\rm{C}}\,{\rm{for}}\,{\rm{i}} = \,1,{\rm{ }}.{\rm{ }}.{\rm{ }}.{\rm{ }},\,{\rm{n and }}\mathop \sum \nolimits^ \left( {{{\rm{\alpha }}_{\rm{i}}}{{\rm{y}}_{\rm{i}}}} \right) = 0} \hfill  \cr 

 } $$

where:

– $\rm \alpha_i$ are the Lagrange multipliers.

– $\rm K(x_i, x_j)$ is the kernel function that maps the input data into a higher-dimensional space.

### KNN

The KNN algorithm is a theoretically mature method and one of the simplest machine learning algorithms. The idea of this method is: in the feature space, if most of the recent k samples near a sample belong to a specific category, then the sample also belongs to this category (Liu et al., 2019). KNN operates on the assumption that similar data points are more likely to belong to the same class or have similar output values. Its primary advantage lies in its ability to adapt to the underlying data distribution, making it suitable for a wide range of applications.

The KNN algorithm consists of the following steps:

1) Choose the number of neighbors, k.
2) Calculate the distance between a query point and all data points in the training set. Common distance measures include Euclidean, Manhattan, and Minkowski distances.
3) Select the k nearest neighbors to the query point based on the calculated distances.
4) For classification, assign the class label that occurs most frequently among the k neighbors to the query point. For regression, assign the average output value of the k neighbors to the query point.

The distance between two data points $\rm x_i$ and $\rm x_j$ can be calculated using the Euclidean distance formula:

$\rm d(x_i, x_j) = \sqrt {\sum\left(\left(x_i - x_j\right)^2\right)}$

where:

– $\rm d(x_i, x_j)$ is the Euclidean distance between points $\rm x_i$ and $\rm x_j$.

– $\rm x_i$ and $\rm x_j$ are data points in a multi-dimensional feature space.

In summary, the K-nearest neighbors algorithm is a straightforward and flexible supervised learning method used for classification and regression. It relies on the principle that similar data points are more likely to have similar output values and calculates the distance between data points to identify the k closest neighbors. The output for a query point is determined by the majority class label or average output value of its k nearest neighbors.

### DT

DT are a popular and interpretable supervised learning method used for classification and regression tasks. DT recursively split the input feature space into regions based on feature values, ultimately leading to a predicted class or continuous output value at the leaf nodes.

It is a graphical method that uses probability analysis intuitively. Supervised learning is given several samples, each with attributes and a category. These categories are determined in advance. Then a classifier is obtained through learning. This classifier can give the object the correct classification (Moreno-Bote & Mastrogiuseppe, 2021). The DT is easy to understand and implement. It can directly reflect the characteristics of the data. If it is explained, it can understand the meaning expressed by the DT. For DT, data preparation is often uncomplicated or unnecessary and can be done simultaneously. Works with both datatype and general-type attributes to produce feasible and effective results on significant data sources in a relatively short period (Patel & Prajapati, 2018; Charbuty & Abdulazeez, 2021; Lee, Cheang & Moslehpour, 2022).

The construction of a DT involves the following steps:

1) Choose a feature and a split point to create a decision node, which will partition the data into two subsets.
2) Calculate the impurity (*e.g*., Gini impurity, entropy) for each subset.
3) Select the feature and split point that result in the largest impurity reduction.
4) Recursively repeat steps 1–3 for each subset until a stopping criterion is met, such as a maximum depth, a minimum number of samples per leaf, or an insignificant impurity reduction.

The impurity reduction can be calculated using the following formula:

$\rm Impurity\, Reduction = I(parent) - \sum((n_{child} / n_{total}) * I(child))$

where:

– I(parent) is the impurity of the parent node.

– $\rm n_{child}$ is the number of samples in the child node.

– $\rm n_{total}$ is the total number of samples in the parent node.

– I(child) is the impurity of the child node.

– The summation is over all child nodes.

In summary, decision trees are a widely-used supervised learning method for classification and regression tasks. They recursively partition the feature space based on feature values, leading to predicted outputs at the leaf nodes. Decision trees are valued for their interpretability and ease of implementation, making them a popular choice for various applications.

## Results

### Feature importance results

**Figure 2:**
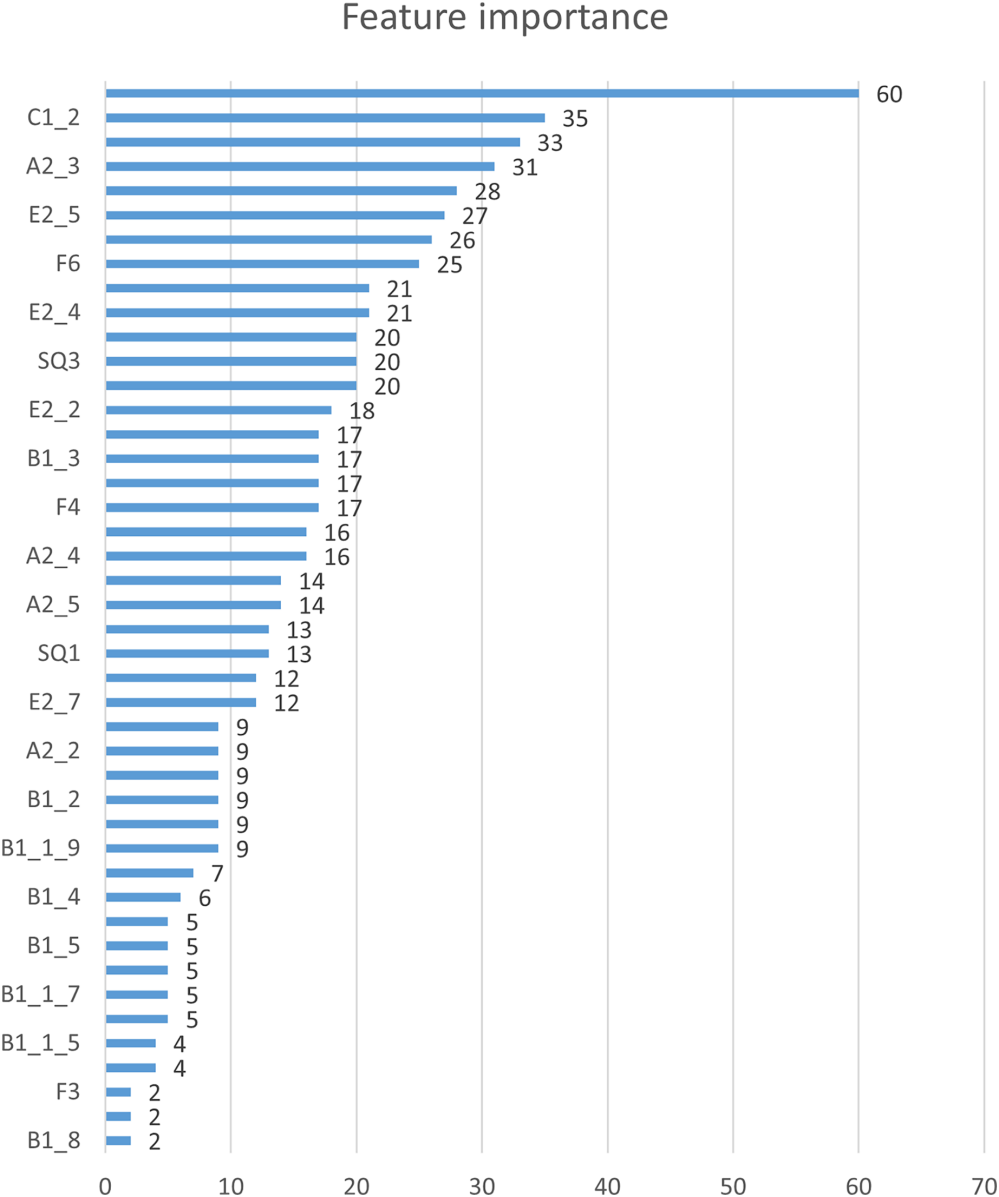
Feature importance.

**Figure 3:**
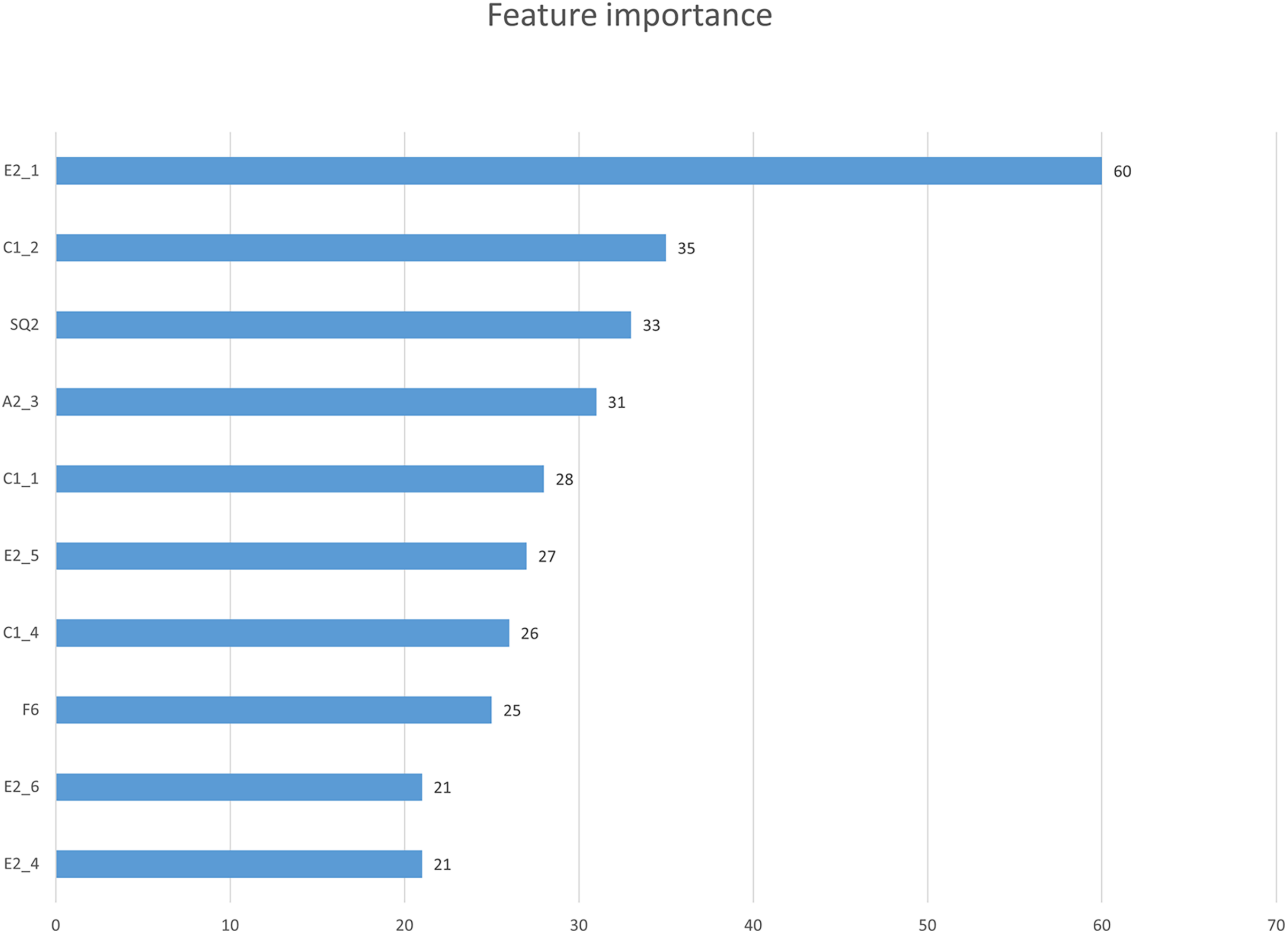
Feature importance scores of 10 key factors.

In this study, we first used the XGBoost feature importance algorithm to obtain scores for all indicators. Figure 2 is the bar chart of the feature importance scores of all features. We then selected the top 10 indicators based on their feature importance scores for the subsequent prediction work. Figure 3 shows a bar chart of the feature importance scores for the 10 indicators, while Table 4 provides detailed information about these indicators. The article classifies the 10 indicators with the highest feature importance scores into four predictable dimensions for predicting user satisfaction with collaboration software. The details are as follows, Institutional and guidance (E2_1, E2_5, E2_6), ICT environment (C1_1, E2_4), Company culture (C1_2, C1_4, A2_3), and demographics (SQ2, F6). The high feature importance scores of the indicators at these levels demonstrate their criticality in predicting employee satisfaction with collaboration software.

**Table 4:** Independent variables.

Ranking	Numbering	Measurement items
1	E2_1	Suppose the government could provide guidance and facilitation laws for companies regarding smart working operations. How much impact will this have on your company’s introduction and use of smart work?
2	C1_2	Is your company’s CEO interested and willing to introduce smart work?
3	SQ2	What industry does the company you work for belong to?
4	A2_3	How much do you think working from home is necessary?
5	C1_1	Your company is well-equipped with the basic environment communication ICT necessary for smart work?
6	E2_5	Suppose the government could provide smart work introduction consulting and advisory support for companies. How much impact will this have on your company’s introduction and use of smart work?
7	C1_4	Your company respects the autonomy of its employees rather than relying on the control or supervision of managers in doing their jobs?
8	F6	What is your position in your company?
9	E2_4	Suppose the government could support the cost required for companies to adopt collaboration software. How much impact will this have on your company's introduction and use of smart work?
10	E2_6	Suppose the government could provide companies with the provision smart work introduction and operation best-case information. How much impact will this have on your company's introduction and use of smart work?
11	SQ4	Company’s sales as of 2019
12	SQ3	How many employees does the company you work for?
13	C1_3	Your company encourages employees to freely use the type of smart work that the company is operating.
14	E2_2	Suppose the government could support building a shared office based on the latest technology in-house for companies. How much impact will this have on your company's introduction and use of smart work?
15	F4	What job do you do within the company?
16	A2_1	How much do you think you need a mobile office?
17	B1_3	Are you using telecommuting?
18	F2	How old are you?
19	A2_4	How much do you think you need a smart office?
20	B1_1_1	When was the first time you used the mobile office
21	A2_5	How much do you think flexible working arrangements are needed?
22	E2_8	Suppose the government could provide an introduction and operation guide for smart work for companies. How much impact will this have on your company's introduction and use of smart work?
23	SQ1	What type of company are you working for?
24	E3	What do you think is the most effective publicity tool that the government should utilize to promote the activation of smart work adoption by enterprises?
25	E2_7	Suppose the government could provide smart work training opportunities for the CEO and smart work department for companies. How much impact will this have on your company’s introduction and use of smart work?
26	B1_1	Are you currently using a mobile office for business purposes?
27	B1_1_9	When was the first time you used the flexible working system?
28	B1_1_4	When was the first time you used flexible seating system?
29	B1_2	Are you using the smart work center?
30	B1_9	Are you using the flexible work system?
31	A2_2	How much do you think smart work centers are needed?
32	F5	Are you a full-time worker? Or is it part-time?
33	F1	what is your gender?
34	B1_4	Are you using the flexible seating system?
35	B1_1_3	When was the first time you used telecommuting?
36	B1_1_7	When was the first time you used staggered commuting system?
37	F7	How long have you been with your current company?
38	B1_5	Are you using video conferencing?
39	B1_1_6	When was the first time you used a messenger for work?
40	B1_1_8	When was the first time you used the discretionary work system?
41	B1_1_5	When was the first time you used video conferencing?
42	B1_8	Are you using the discretionary work system?
43	B1_7	Are you using the staggered commuting system?
44	F3	Do you have children under elementary school age to be cared for?

### Prediction accuracy results

In this study, we focused on relevant data related to critical indicators presented in Fig. 3 to evaluate the prediction accuracy of various machine learning-based binary classifiers. The prediction accuracy of these classifiers is presented in Table 5. Our analysis indicated that the NB classifier achieved the highest prediction accuracy of 0.780, followed by LR (0.767), XGBoost (0.744), SVM (0.744), KNN (0.704), and DT (0.637). Therefore, the NB classifier is the preferred predictive model for employee satisfaction with collaboration software in this study. This result further demonstrates the numerous advantages of NB classifiers in predicting panel data. For instance, it is simple and efficient, capable of handling missing and noisy data, and exhibits good interpretability.

**Table 5:** Prediction accuracy.

Binary classifiers	Accuracy
NB	0.780
LR	0.767
XGB	0.744
SVM	0.744
KNN	0.707
DT	0.637

## Discussion

Based on the results of the feature importance analysis, our research has identified four dimensions that can predict organizational members’ satisfaction with collaboration software: institutional guidance, ICT environment, company culture, and demographics. These dimensions have practical implications for enterprises in improving user satisfaction.

Firstly, the establishment of sound institutional guidance is critical in predicting user satisfaction. Enterprises can build a stable and standardized basic institutional guidance to use collaboration software, including specific workflows, timing, and expected outcomes for employees.

Secondly, a complete ICT environment is essential for predicting user satisfaction. Enterprises can check the ICT environment to confirm whether an upgrade plan for the information system is necessary. Moreover, companies can improve the compensation system to ease the financial burden on employees.

Thirdly, company culture and demographics are crucial factors in predicting user satisfaction. The enthusiasm and will of top management can encourage employees to embrace the new system, and leaders can motivate the individual and the entire team.

Lastly, our research has contributed to the forecasting research field by focusing on the stage before the use of collaboration software, bridging the gap in traditional research methods.

Based on the results of prediction accuracy, our analysis indicates that the machine learning-based NB classifier is the most suitable predictive model for user satisfaction with collaboration software. This model has demonstrated excellent performance in panel data analysis and outperformed the other algorithms considered in this study. One of the advantages of the NB classifier is its simplicity and computational efficiency, which makes it easy to implement. The NB classifier can estimate the posterior probability of each class, providing interpretable results for understanding the factors that contribute to user satisfaction with collaboration software. This interpretability is important for organizations to identify the most critical factors that affect user satisfaction and formulate targeted strategies for improvement.

In contrast, other classifiers have their own shortcomings when predicting panel data. For instance, LR may assume a linear relationship between predictor variables and outcomes, which may not be appropriate for complex non-linear relationships. XGBoost may require careful tuning of hyperparameters and can be more difficult to interpret than other algorithms, despite its ability to handle different data types and perform well in many contexts. SVM may require a large amount of computational resources and be sensitive to the choice of kernel function and parameters. KNN may be computationally intensive, especially for large datasets, and require careful tuning of hyperparameters to achieve optimal performance. DT may overfit when there are many predictor variables or when the tree is allowed to grow too deep and may not perform well when there are complex relationships between predictor variables.

In conclusion, our findings suggest that the NB classifier is a promising predictive model for user satisfaction with collaboration software, particularly in the context of panel data analysis.

## Conclusion

The complexity of accurately predicting future outcomes in business forecasting has long posed challenges for researchers. In response, this study employed a feature importance algorithm to extract key variables from intricate data sets and utilized a machine learning-based binary classifier to validate the prediction accuracy of these variables.

By predicting user satisfaction prior to introducing collaboration software, businesses can proactively identify and resolve potential issues, thereby enhancing user adoption and minimizing resistance to change. This approach also enables companies to avoid investing in software that might not meet their employees’ needs, ultimately reducing costs related to software acquisition and implementation.

In conclusion, our research demonstrates that predicting user satisfaction with collaboration software plays a vital role in supporting businesses as they pursue their digital transformation goals. By fostering improved collaboration, communication, and knowledge sharing among employees, this study contributes to the development of more effective strategies for implementing new technologies and propelling organizational success.

The applicability of our findings to the industry is evident in the potential for innovation and improved decision-making processes. By employing machine learning-based classifiers to accurately predict user satisfaction, companies can make more informed choices about collaboration software selection, ultimately streamlining their digital transformation journey. Furthermore, our proposed approach is easily adaptable to various industry contexts, as it relies on machine learning techniques that can be fine-tuned to suit the specific requirements of diverse organizations.

The implications of our research extend beyond mere cost savings; by facilitating more effective collaboration and communication among employees, companies can experience increased productivity, enhanced innovation, and overall organizational growth. By adopting the methods outlined in this study, businesses can create a more harmonious and efficient work environment, ultimately leading to a competitive advantage in today's rapidly evolving digital landscape.

### Limitations and future research

While this study has provided insights into business management and forecasting, it is important to acknowledge that the timing of the data collection may present some limitations. The data were collected during the COVID-19 pandemic when many people were working remotely and using collaboration software to prevent the spread of the virus. This unique situation may have influenced user satisfaction with the software. As we move towards a post-pandemic era, it is possible that important indicators for predicting user satisfaction with collaboration software may shift, making it necessary to continue collecting relevant data and conducting predictive analyses.

## Supplemental Information

10.7717/peerj-cs.1481/supp-1 Supplemental Information 1 Raw data.

## Additional Information and Declarations

### Data Availability

The following information was supplied regarding data availability: The data is available at Zenodo: YITUO FENG. (2022). FYTClimbing/predicting-satisfaction-with-collaboration-software: v1.0.0 (v1.0.0). Zenodo. https://doi.org/10.5281/zenodo.7187677.
